# Supplementary figures and images for: Investigation of the therapeutic efficacy and resistance mechanisms of lytic phages targeting ST218 KL57 CR-hvKP
Source: mSystems. 2026 Jan 16;11(2):e01476-25. doi: 10.1128/msystems.01476-25 (PMC12911405; doi:10.1128/msystems.01476-25)

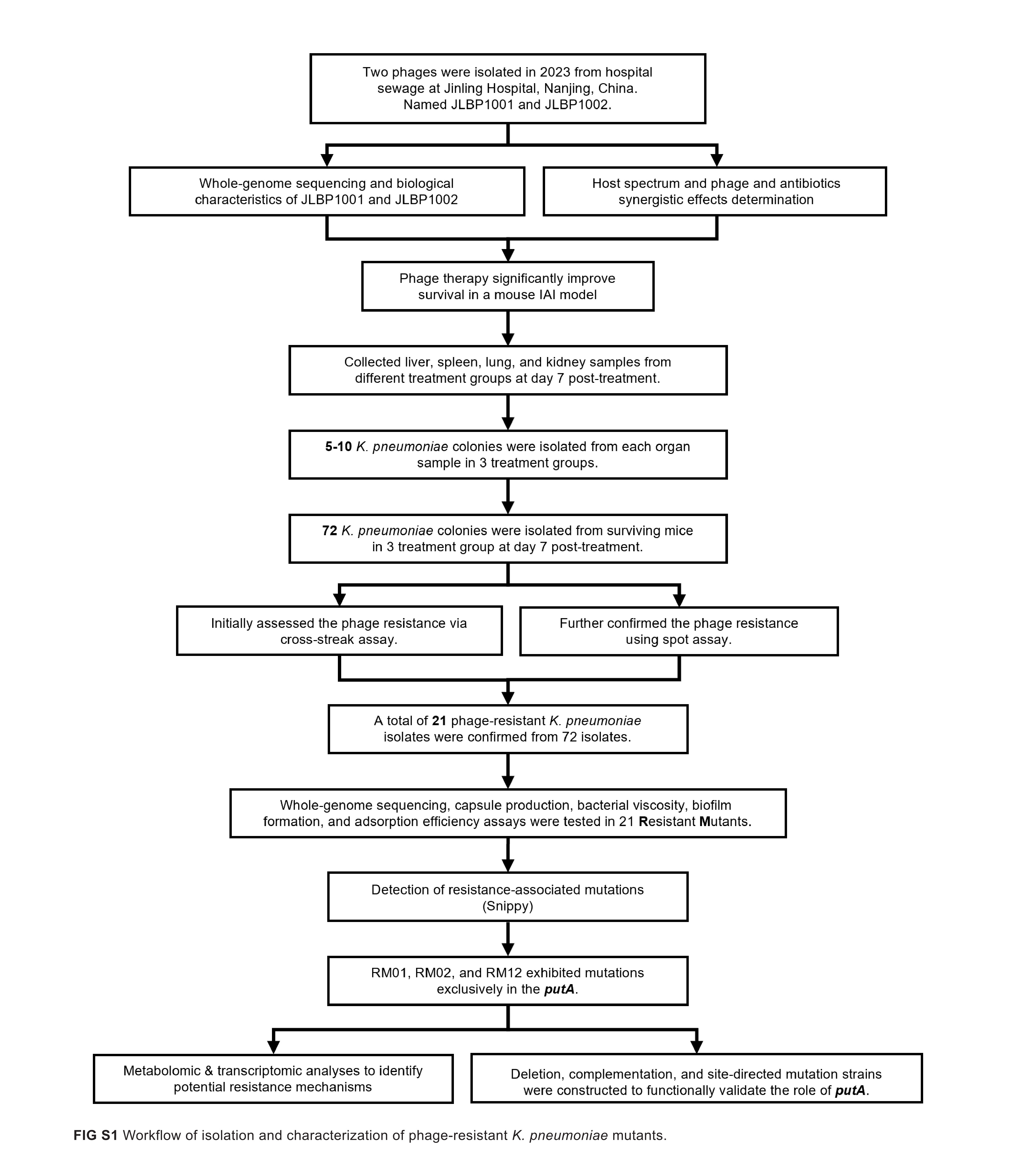

Supplement: Fig. S1 — Workflow of isolation and characterization of mutants. [file msystems.01476-25-s0001.tif]

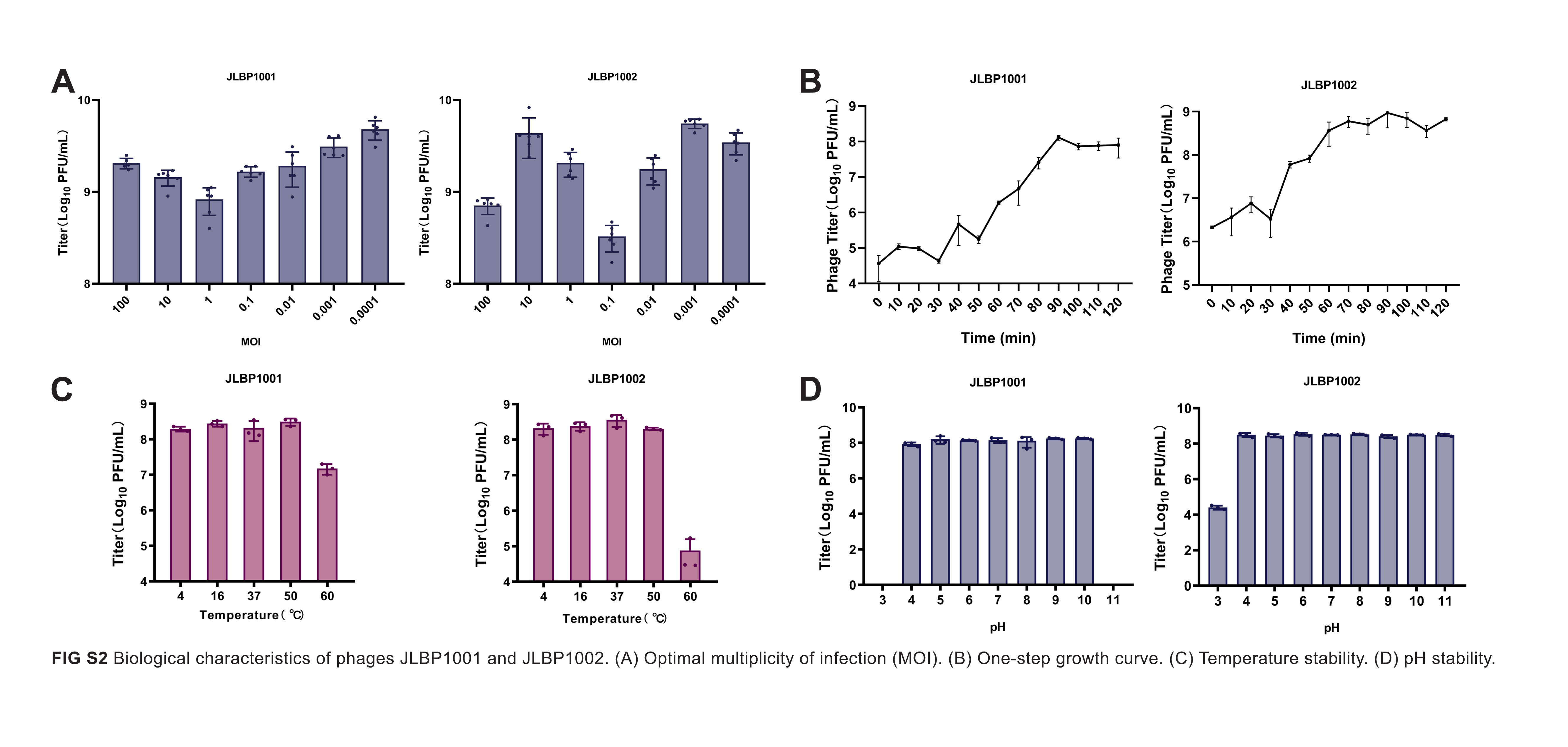

Supplement: Fig. S2 — Biological characteristics of JLBP1001 and JLBP1002. [file msystems.01476-25-s0002.tif]

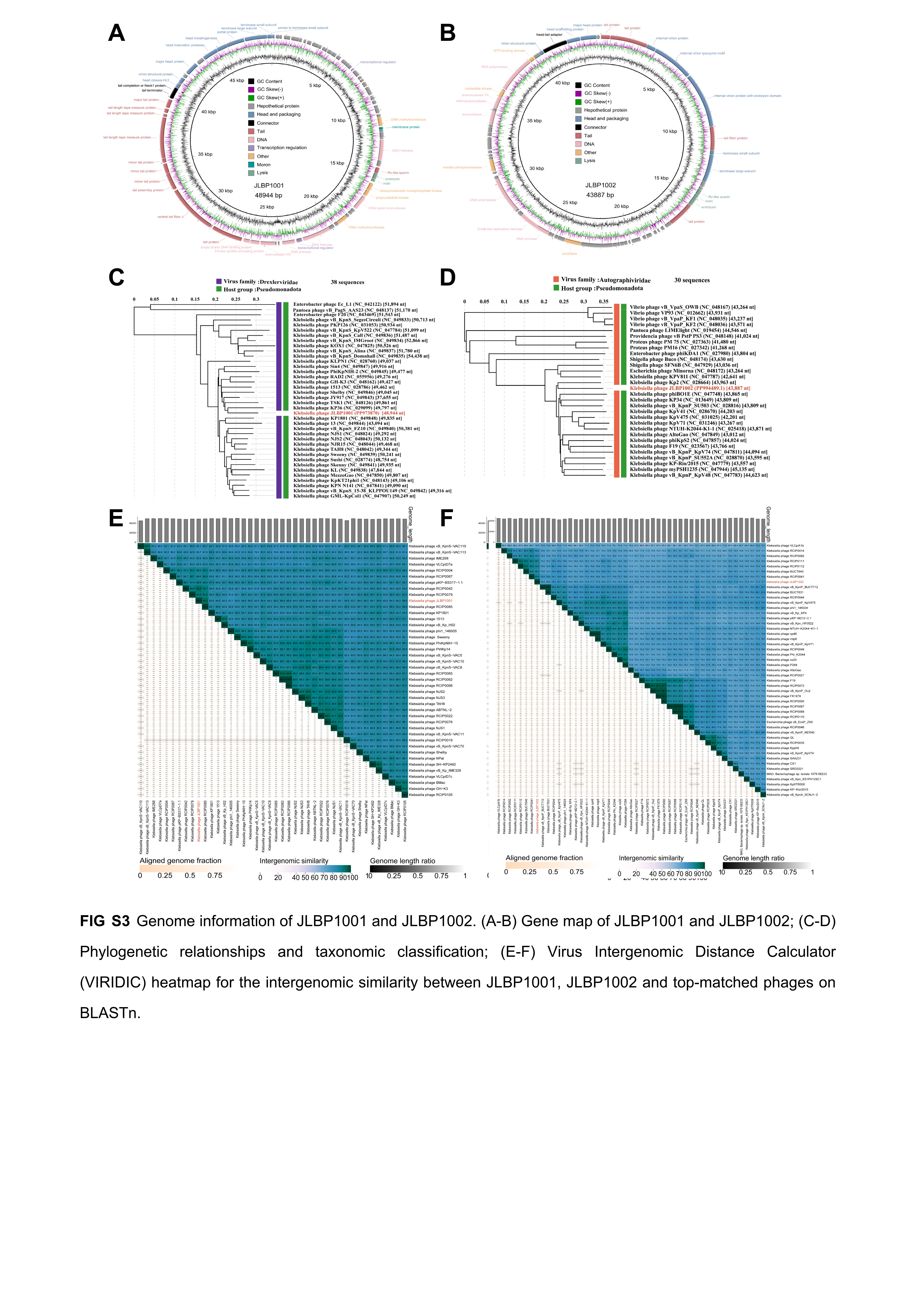

Supplement: Fig. S3 — Genome information for JLBP1001 and JLBP1002. [file msystems.01476-25-s0003.tif]

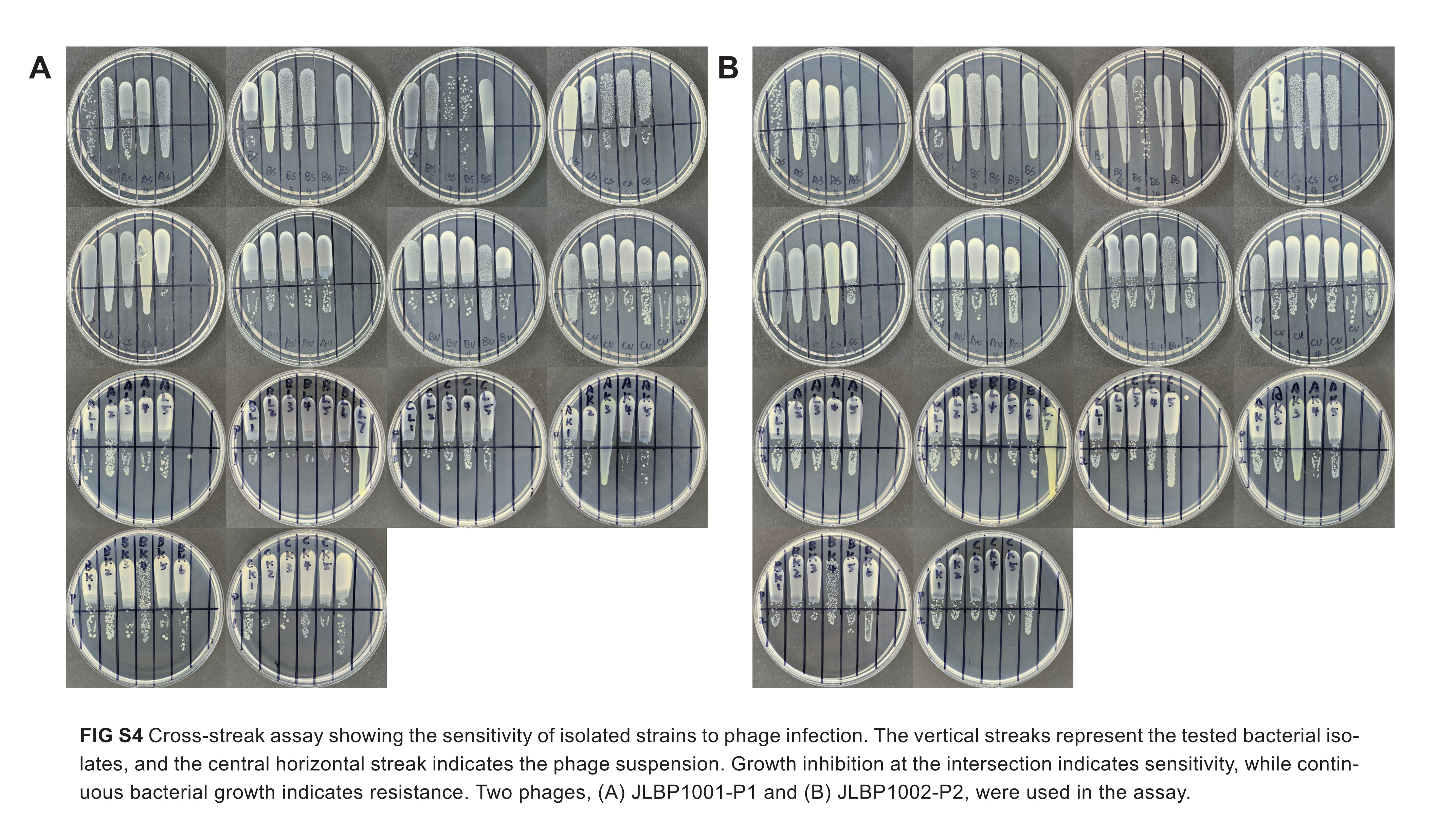

Supplement: Fig. S4 — Sensitivity of isolated strains to phage infection. [file msystems.01476-25-s0004.tif]

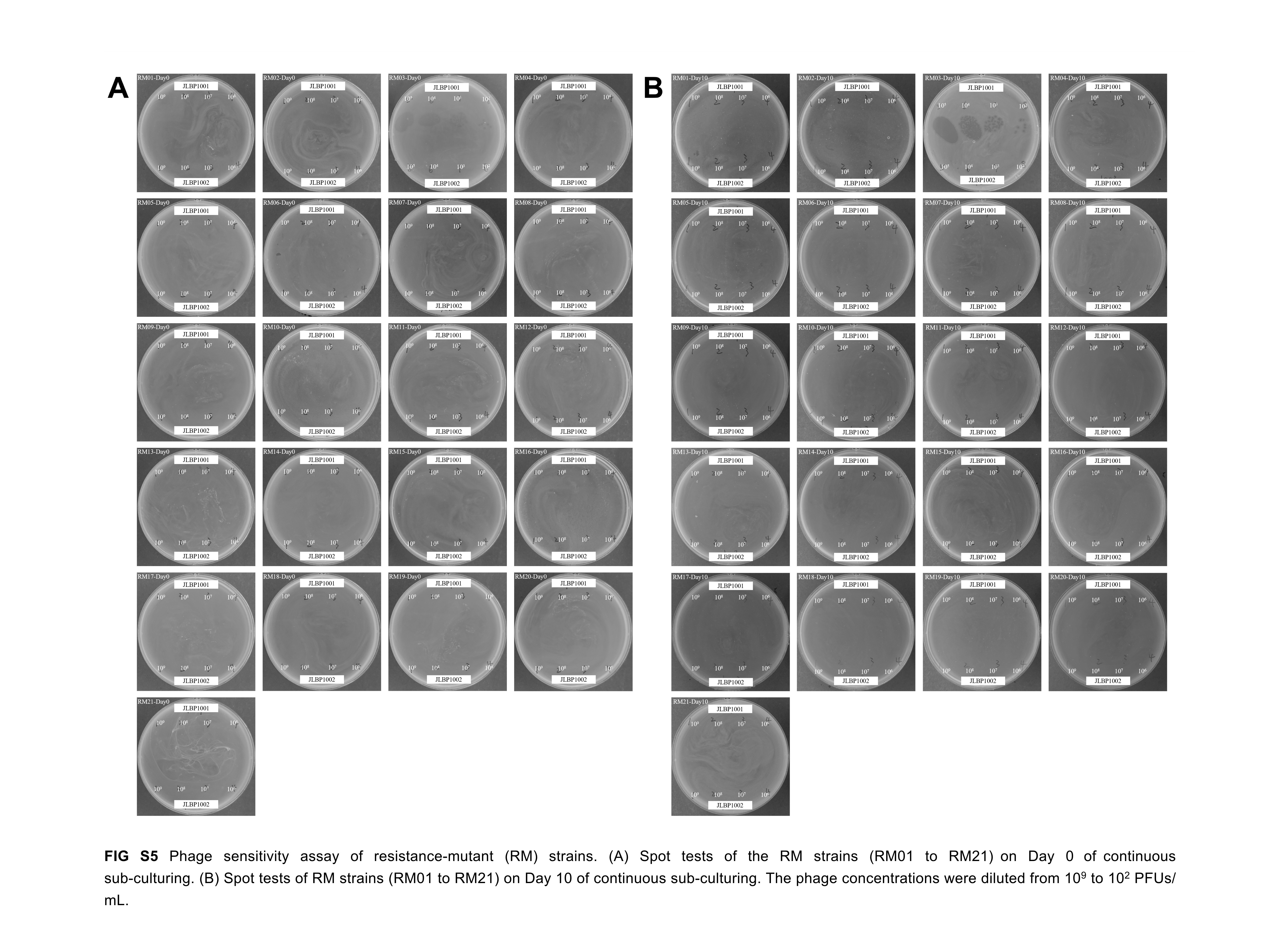

Supplement: Fig. S5 — Phage sensitivity assay of RM strains. [file msystems.01476-25-s0005.tif]

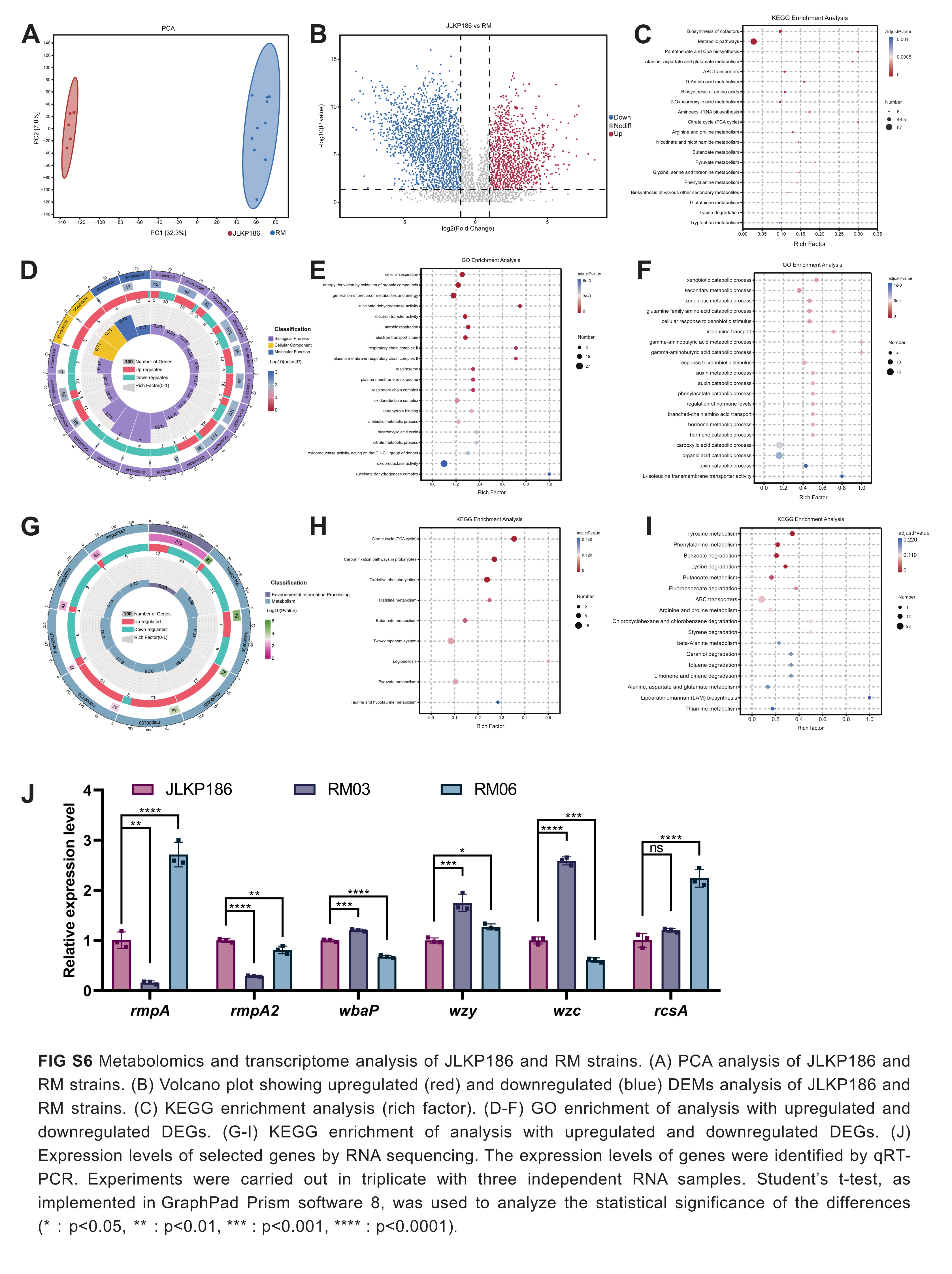

Supplement: Fig. S6 — Metabolomics and transcriptome analysis of JLKP186 and RM strains. [file msystems.01476-25-s0006.tif]
