# Supplementary material for: Investigation of the therapeutic efficacy and resistance mechanisms of lytic phages targeting ST218 KL57 CR-hvKP
Source: mSystems. 2026 Jan 16;11(2):e01476-25. doi: 10.1128/msystems.01476-25 (PMC12911405; doi:10.1128/msystems.01476-25)
Supplement: Supplemental Tables — Tables S1 to S4. [file msystems.01476-25-s0008.docx]

**Investigation of** **the therapeutic efficacy and resistance mechanisms of lytic phages targeting ST218 KL57 CR-hvKP**

Liuqing Dou,^1^* Jiayang Li,^2^*^,#^ Wenqi Wu,^2^ Li Xu,^3^ Mingjie Qiu,^3^ Shuanghong Yang,^1^ Jiajie Wang,^4^ Sai Tian,^1^ Zhitao Zhou,^3^ Meilin Wu,^3^ Yun Zhao,^5^ Xiuwen Wu,^1 2#^ & Jianan Ren,^1 2#^

^1^Jinling Clinical Medical College, Nanjing University of Chinese Medicine, Nanjing, China

^2^Research Institute of General Surgery, Jinling Hospital, the Affiliated Hospital of Medical School, Nanjing University, Nanjing, China

^3^Research Institute of General Surgery, Jinling Hospital, the Affiliated Hospital of Medical School, Nanjing Medical University, Nanjing, China

^4^Research Institute of General Surgery, Jinling Hospital, School of Medicine, Southeast university, Nanjing, China

^5^Clinical Translational Research Center for Surgical Infection and Immunity of Nanjing Medical University, Nanjing, China

*These authors contributed equally to this work: Liuqing Dou, Jiayang Li. The author order was determined in order of increasing seniority.

These authors jointly supervised this work: Xiuwen Wu & Jianan Ren.

#Correspondence authors

Jiayang Li, PhD, Research Institute of General Surgery, Jinling Hospital, the Affiliated Hospital of Medical School, Nanjing University, 305 East Zhongshan Road, Nanjing 210009, China. Telephone: +86-25-80863217. E-mail: jiayanglinj@163.com

Xiuwen Wu, Professor, Jinling Clinical Medical College, Nanjing University of Chinese Medicine, 305 East Zhongshan Road, Nanjing 210002, P.R. China. E-mail addresses: wuxiuwen@nju.edu.cn

Jianan Ren, Professor, Jinling Clinical Medical College, Nanjing University of Chinese Medicine, 305 East Zhongshan Road, Nanjing 210002, P.R. China. E-mail addresses: jiananr@nju.edu.cn

**Table S1** Antimicrobial drug susceptibility test of *K. pneumoniae* JLKP186 and RM strains.

| **Strains ID** | **MICs(mg/L)** | | | | | | | | |
| --- | --- | --- | --- | --- | --- | --- | --- | --- | --- |
|  | **TCC** | **TZP** | **CAZ** | **CSL** | **FEP** | **ATM** | **IPM** | **MEM** | **CAZ-AVI** |
| JLKP186 | >128/R | >128/R | 32/R | >64/R | >32/R | >64/R | 16 R | 16 R | 2/4 S |
| RM01 | >128/R | >128/R | 32/R | >64/R | >32/R | >64/R | 16/R | 32 R | 2/4 S |
| RM02 | >128/R | >128/R | 32/R | >64/R | >32/R | >64/R | 32 R | 32 R | 4/4 S |
| RM03 | >128/R | >128/R | 32/R | >64/R | >32/R | >64/R | 16/R | 32 R | 4/4 S |
| RM04 | >128/R | >128/R | 32/R | >64/R | >32/R | >64/R | 16/R | 32 R | 2/4 S |
| RM05 | >128/R | >128/R | 32/R | >64/R | >32/R | >64/R | 32 R | 32 R | 2/4 S |
| RM06 | >128/R | >128/R | 32/R | >64/R | >32/R | >64/R | 32 R | 32 R | 2/4 S |
| RM07 | >128/R | >128/R | 32/R | >64/R | >32/R | >64/R | 16/R | 32 R | 2/4 S |
| RM08 | >128/R | >128/R | 32/R | >64/R | >32/R | >64/R | 16/R | 16/R | 1/4 S |
| RM09 | >128/R | >128/R | 32/R | >64/R | >32/R | >64/R | 16/R | 16/R | 2/4 S |
| RM10 | >128/R | >128/R | 32/R | >64/R | >32/R | >64/R | 64/R | 16/R | 2/4 S |
| RM11 | >128/R | >128/R | 32/R | >64/R | >32/R | >64/R | 32 R | 32 R | 2/4 S |
| RM12 | >128/R | >128/R | 32/R | >64/R | >32/R | >64/R | 32 R | 32 R | 2/4 S |
| RM13 | >128/R | >128/R | 32/R | >64/R | >32/R | >64/R | 64/R | 32 R | 2/4 S |
| RM14 | >128/R | >128/R | 32/R | >64/R | >32/R | >64/R | 32 R | 16/R | 2/4 S |
| RM15 | >128/R | >128/R | 32/R | >64/R | >32/R | >64/R | 32 R | 16/R | 1/4 S |
| RM16 | >128/R | >128/R | 32/R | >64/R | >32/R | >64/R | 32 R | 16/R | 4/4 S |
| RM17 | >128/R | >128/R | 32/R | >64/R | >32/R | >64/R | 16/R | 32 R | 2/4 S |
| RM18 | >128/R | >128/R | 32/R | >64/R | >32/R | >64/R | 16/R | 32 R | 2/4 S |
| RM19 | >128/R | >128/R | 32/R | >64/R | >32/R | >64/R | 32 R | 16/R | 1/4 S |
| RM20 | >128/R | >128/R | 32/R | 32 I | 32/R | >64/R | 32 R | 16/R | 1/4 S |
| RM21 | >128/R | >128/R | 32/R | >64/R | >32/R | >64/R | 64/R | 64/R | 2/4 S |

Abbreviations: MIC, minimum inhibitory concentration; S, susceptible; R, resistant; TCC, ticarcillin–clavulanate; TZP, piperacillin–tazobactam; CAZ, ceftazidime; CSL, cefoperazone–sulbactam; FEP, cefepime; ATM, aztreonam; IPM, imipenem; MEM, meropenem; CAZ-AVI, ceftazidime–avibactam.

**Table S2** Clinical strains used in the host range of JLBP1001 and JLBP1002.

| **Strains** | **Year** | **ST** | **Capsule locus** | **Source** |
| --- | --- | --- | --- | --- |
| A36 | 2020 | 11 | KL47 | Tissue |
| A41  A48  B127 | 2020  2020  2020 | 2286  29  11 | KL10  KL54  KL15 | Alveolar lavage fluid  Sputum  Ascites |
| B134 | 2020 | 11 | KL10 | Alveolar lavage fluid |
| B139  B161  B208 | 2020  2020  2020 | 1027  11  29 | KL20  KL15  KL54 | Blood  Blood  Ascites |
| B211 | 2020 | 11 | KL47 | Blood |
| B215 | 2020 | 11 | KL47 | Alveolar lavage fluid |
| B231 | 2020 | 11 | KL47 | Bile |
| B233 | 2020 | 15 | KL112 | Blood |
| B235 | 2020 | 11 | KL47 | Blood |
| B236 | 2020 | 2237 | KL19 | Blood |
| B237 | 2020 | 11 | KL10 | Ascites |
| B240 | 2020 | 11 | KL25 | Ascites |
| B256 | 2020 | 617 | KL122 | Pancreatic juice |
| B261 | 2020 | 11 | KL64 | Bile |
| B269 | 2020 | 15 | KL19 | Pleural effusion |
| B317 | 2020 | 11 | KL25 | Pancreatic juice |
| B327 | 2020 | 11 | KL25 | Alveolar lavage fluid |
| D004 | 2020 | 111 | KL63 | Pleural effusion |
| D007 | 2020 | 11 | KL25 | Bile |
| D016 | 2020 | 5422 | KL21 | Ascites |
| D023 | 2020 | 17 | KL2 | Ascites |
| D025 | 2020 | 15 | KL112 | Ascites |
| D053 | 2020 | 320 | KL9 | Pancreatic juice |
| D059 | 2020 | 15 | KL112 | Pancreatic juice |
| D066 | 2020 | 15 | KL112 | Ascites |
| D067 | 2020 | 15 | KL19 | Ascites |
| D070 | 2020 | 11 | KL64 | Alveolar lavage fluid |
| D073 | 2020 | 11 | KL30 | Pancreatic juice |
| D077 | 2020 | 11 | KL64 | Pleural effusion |
| D087 | 2020 | 11 | KL64 | Ascites |
| D096 | 2020 | 11 | KL10 | Ascites |
| D098 | 2020 | 11 | KL30 | Pancreatic juice |
| D102 | 2020 | 11 | KL30 | Pancreatic juice |
| D103 | 2020 | 2237 | KL19 | Ascites |
| D111 | 2020 | 11 | KL30 | Ascites |
| D112 | 2020 | 11 | KL64 | Ascites |
| JLKP186 | 2023 | 218 | KL57 | Ascites |
| K300 | 2023 | 11 | KL54 | Bile |
| N591 | 2023 | 592 | KL57 | Farm sewage |
| N947 | 2024 | 11 | KL57 | Farm sewage |
| N949  NTUH-2044  ATCC13883  ATCC43816 | 2024 | 11 | KL57 | Farm sewage  Reference strain  Reference strain  Reference strain |

**Table S3** Primers of gene *putA* in cloning and complementation experiments.

| **Primer** | **Sequence (5’ - 3’)** |
| --- | --- |
| *putA*-sgRNA | AATTCGAGGAACGGCTGATA |
| *putA*-up-F | CAGCGTCAGGGCGTTATTATTCA |
| *putA*-up-R | CCACCGGGGAGCCCGTTGCTCAAAACTCCTGTCATTTATC |
| *putA*-down-F | ATGACAGGAGTTTTGAGCAACGGGCTCCCCGGTGGCGCTA |
| *putA*-down-R | CGCCGGTCGATCCACAGCATTTA |
| *putA*-19-F | TGTAAAACGACGGCCAGTCAGCGTCAGGGCGTTATTATTCA  CTATGACCATGATTACGCCGGTCGATCCACAGCATTTA |
| *putA*-19-R |  |
| *putA*-Ter-F | GCCGCGGCATTTATGAAGGTC |
| *putA*-Ter-R  *putA*-AprF  *putA*-AprF  *putA*-mut-JD-F  *putA*-CX-R | ACATCACGCCGCGTACGCGCTCA  TGAACTGCATGAATTCCCAGCTTTACCCGCGGCTGAAATCG  TGAACTGCATGAATTCCCGGCTTCGTGATTCAGGCC  CGAACCGCTGTACGAGCAGGTGG  ACATCACGCCGCGTACGCGCTCA |

**Table S4** Primers of gene *putA*, *putP*, CPS gene cluster, and transcriptional regulators.

| **Primer** | **Sequence (5’ - 3’)** |
| --- | --- |
| *rmpA*-F | AATGTTAGCCGAATTGTAAACCA |
| *rmpA*-R | CCCCCATTTTTCAGTAGGCAT |
| *rmpA2*-F | AAGGCTCGATGGATAAACCAA |
| *rmpA2*-R | TCAAGCCACATCCATTGACT |
| *wbaP*-F | ATTTCTCCCGCTATGTTTGGT |
| *wbaP*-R | AACCCCATTTCATCAACACCC |
| *wzy*-F | ACGATTTTCAACTTGGGCTT |
| *wzy*-R | ACCCAATTATTCCCGTTTCTACC |
| *putA*-F | AAAACCTTCAGCAACGCCATC |
| *putA-R* | TGATACAGACCACCGGACCC |
| *putP*-F | GCCAGCCTGATGATTTTCGC |
| *putP*-R | AGCCCTTTCAGCATGTCGAT |
